# Molecular differentiation of *Opisthorchis felineus* and *Metorchis bilis* in cyprinid fish from Northern Kazakhstan: Epidemiological insights and diagnostic advances

**DOI:** 10.14202/vetworld.2025.3197-3207

**Published:** 2025-10-31

**Authors:** Aiganym Bekaidarovna Bekenova, Aleksey V. Katokhin, Kundyz B. Muratbekova

**Affiliations:** 1 Department of Microbiology and Biotechnology, S. Seifullin Kazakh Agro Technical Research University, Astana, Kazakhstan; 2 Department of Department of Insect Genetics, Institute of Cytology and Genetics, Siberian Branch of the Russian Academy of Sciences, Novosibirsk, Russia

**Keywords:** cytochrome c oxidase subunit 1, Kazakhstan, metorchiasis, *Metorchis bilis*, multiplex polymerase chain reaction, opisthorchiasis, *Opisthorchis felineus*, zoonotic trematodes

## Abstract

**Background and Aim:**

Opisthorchiasis and metorchiasis are significant zoonotic fish-borne trematodiases caused by *Opisthorchis felineus* and *Metorchis bilis*. These parasites exhibit overlapping geographic ranges and morphologically similar larval stages, complicating species-level identification. In Kazakhstan, where raw or undercooked freshwater fish is widely consumed, opisthorchiasis remains an endemic concern. This study aimed to investigate the prevalence of *O. felineus* and *M. bilis* in cyprinid fish from Akmola Region and to establish molecular tools for their differential diagnosis.

**Materials and Methods:**

A total of 818 freshwater cyprinid fish were collected from Lakes Sholak, Esey, and Karazhar between 2021 and 2023. Muscle tissue was examined using the compression method for metacercariae detection. Morphological identification was complemented with a newly designed multiplex polymerase chain reaction (PCR) assay targeting the mitochondrial cytochrome c oxidase subunit 1 (*COX1*) gene. Selected amplicons were sequenced and subjected to phylogenetic analysis. Prevalence and infection intensity were calculated, and statistical comparisons were made among fish species and lakes.

**Results:**

*Opisthorchiidae* metacercariae were detected in ide, bream, and roach, with prevalence varying across lakes. Lake Sholak exhibited the highest infection rate (42.9%), with ide showing the greatest susceptibility (40.4%). No infections were detected in fish from Lake Karazhar. Morphological differentiation between *O. felineus* and *M. bilis* was inconclusive due to overlapping features. Multiplex PCR successfully distinguished *O. felineus* (307 bp) from *M. bilis* (252 bp), with >99% sequence identity to GenBank references. Two representative sequences (PQ669120 and PQ669125) were deposited in GenBank. Phylogenetic analysis confirmed distinct clustering of both species.

**Conclusion:**

This study provides the first molecular confirmation of *O. felineus* and *M. bilis* in freshwater fish of the Akmola region, Kazakhstan. The developed multiplex PCR assay offers a sensitive and reliable diagnostic tool for species-level differentiation. These findings highlight moderate to high prevalence in local fish, underline the zoonotic risks associated with fish consumption, and emphasize the need for integrated One Health surveillance to inform control strategies and food safety policies.

## INTRODUCTION

*Opisthorchis felineus* (*O. felineus*) and *Metorchis bilis* (*M. bilis*), belonging to the family *Opisthorchiidae*, represent a significant concern in both veterinary and human medicine, ranking 8^th^ on the global list of 24 clinically significant foodborne parasites [1]. The life cycle of the *Opisthorchiidae* family involves two intermediate and one definitive host. Freshwater snails of the genus *Bithynia* act as the first intermediate hosts of liver flukes in this family. Within these mollusks, the parasites undergo essential developmental stages from miracidia to cercariae, highlighting their pivotal role in sustaining transmission and maintaining the natural foci of opisthorchiasis [2]. The geographical distribution and ecological characteristics of *Bithynia* spp. strongly influence the endemicity and epidemiological patterns of opisthorchiasis, particularly in the Ob–Irtysh river basin and other regions of Siberia [3]. Snails serve as the first intermediate host, enabling transmission to freshwater fish, which are then consumed by humans and other definitive hosts. Consumption of raw or marinated fish filets without proper heat treatment can result in infection with *O. felineus* and *M. bilis* larvae [4].

In endemic regions, the infection rate of ide with *O. felineus* reaches up to 100%, and in tench, it reaches up to 95% [5]. *O. felineus* is part of the triad of epidemiologically significant liver flukes and is the primary causative agent of opisthorchiasis [6]. *M. bilis* belongs to the genus *Metorchis* and is the causative agent of metorchiasis. The geographic distribution ranges of *O. felineus* and *M. bilis* overlap and extend from the Iberian Peninsula to Eastern Europe and Western Siberia [7]. In Kazakhstan, opisthorchiasis remains a significant natural focal parasitic infection associated with the consumption of freshwater fish containing infective parasite stages. Fishing practices, traditional dietary habits, and methods of fish preparation contribute to the maintenance of high levels of human infection in endemic regions. According to the National Center for Public Health of the Republic of Kazakhstan, the incidence of opisthorchiasis in certain regions varies from 30 to 120 cases/100,000 people. The most affected regions remain Pavlodar, East Kazakhstan, and North Kazakhstan [8]. According to regional health authorities, in recent years, outbreaks have also been recorded in West Kazakhstan, where in 2023, more than 100 cases were registered, representing a 26% increase compared to the previous year. In Astana, 45 cases of opisthorchiasis were reported in 2023 (3.2/100,000 population), whereas 108 cases were reported in 2022 (8.0/100,000 population). Epidemiological monitoring data also confirm a wide prevalence of *O. felineus* larvae among cyprinid fish in several regions of Kazakhstan, indicating the persistence of active natural foci of infection [9]. According to various data, approximately 14 million people worldwide are infected, and 300 million are at risk of infection with members of the *Opisthorchiidae* family [10].

Symptoms and disease severity are closely associated with infection intensity and duration. Because both pathogens exhibit similar morphological features at the larval stage, species-level identification and differentiation of the specific causative agents are difficult. In Western European countries, such as the United Kingdom and France, as well as in North America (the United States and Canada), the diagnosis of metorchiasis is rarely made largely because this disease is infrequently included in clinical diagnostic practices [11–13]. In Kazakhstan, the treatment of opisthorchiasis is included in the clinical protocol of the Ministry of Health of the Republic of Kazakhstan under code “ICD-10: B66.0 Opisthorchiasis” dated November 20, 2015, Protocol No. 16. However, the diagnosis and treatment of metorchiasis are not covered by legal or regulatory documents. In cases of chronic helminthiasis, adult *O. felineus* worms parasitize the liver, causing opisthorchiasis, whereas *M. bilis* localizes in the gallbladder and bile ducts, causing metorchiasis [14].

In veterinary and medical practice, coproscopy and duodenal intubation are the standard methods for diagnosing opisthorchiasis [15]. However, the cyclical nature of helminth egg production and the uneven distribution of eggs in the large intestine often lead to false-negative results. Immunodiagnostics is used as an additional diagnostic method, but it demonstrates high diagnostic value only during the acute phase of opisthorchiasis, while in chronic cases, an immune response is detected in only about 70% of infections. Furthermore, the antigenic similarity between opisthorchis and metorchis may result in false-positive outcomes in mixed infections. Molecular genetic methods can address these diagnostic challenges, as polymerase chain reaction (PCR) enables the detection of *O. felineus* and *M. bilis* at any developmental stage (egg, miracidium, metacercaria, or adult worm).

Therefore, the development of highly sensitive and specific PCR assays for *Opisthorchiidae* identification is of great importance. To date, species-specific primers targeting the internal transcribed spacer (ITS2) region of the nuclear ribosomal gene have been developed in Germany, demonstrating the ability to selectively amplify DNA fragments of several *Opisthorchiidae* species, including *O. felineus*, *Clonorchis sinensis*, *Opisthorchis viverrini*, *Metorchis xanthosomus*, and *Ploesoma truncatum* [16]. Pauly *et al*. [17] synthesized primers targeting the mitochondrial *COX1* gene for *O. felineus* and *M. bilis*, confirming that this gene region serves as a reliable molecular marker for the family. In Russia, PCR assays based on the ITS2 genetic marker have been used to detect mixed infections of *O. felineus* and *M. bilis* in human samples [18]. Moreover, differences in the gene sequences of *Opisthorchiidae* parasites have been observed across different regions of the world. Nucleotide substitutions, predominantly A–G and T–C transitions, have been recorded, although they do not always lead to amino acid changes. Most substitutions are non-synonymous, suggesting adaptation or geographic separation of populations. For example, in northern Jutland, analysis of mitochondrial *COX1* and *COX3* genes revealed two monophyletic clades with four distinct *M. bilis* haplotypes among Danish otters [19]. Similarly, four *COX1* haplotypes differing by 5–7 nucleotides (~1.1% variation across 650 bp) have been identified in Western Siberia and the Volga region [20].

Thus, the development of highly sensitive methods for the differential diagnosis of *O. felineus* and *M. bilis* from various geographic regions using modern molecular techniques is necessary to expand knowledge of these pathogens, achieve precise identification, and inform treatment strategies. Published molecular data on fish-borne helminthiases in Kazakhstan remain scarce. Accurate and reliable differential diagnosis between *O. felineus* and *M. bilis* is essential for effective surveillance and control strategies under the “One Health” framework, as these parasites affect humans, fish, and domestic and wild animals [21]. Sustainable development goals also emphasize improved diagnostics and surveillance of neglected zoonoses, including opisthorchiasis. Nevertheless, epidemiological studies combining field surveillance with molecular identification of *Opisthorchiidae* species in Kazakhstan are still limited [22].

Although *O. felineus* and *M. bilis* are recognized as significant foodborne zoonotic parasites of the family *Opisthorchiidae*, challenges remain in their accurate identification and surveillance. The overlapping geographical distribution of *O. felineus* and *M. bilis* across Eurasia, combined with the morphological similarity of their larval stages, makes reliable species-level diagnosis extremely difficult using conventional parasitological and immunological techniques. In Kazakhstan, opisthorchiasis continues to represent a major public health concern, with prevalence rates ranging from 30 to 120 cases/100,000 population and outbreaks being reported in multiple regions, including Pavlodar, East Kazakhstan, North Kazakhstan, and recently West Kazakhstan. Despite this burden, the diagnosis and treatment of metorchiasis are not addressed by existing regulatory protocols, leading to an underestimation of its clinical and epidemiological impact. Furthermore, reliance on coproscopy, duodenal intubation, and immunodiagnostics often results in false-negative or false-positive outcomes, particularly in mixed infections, due to antigenic similarity between *Opisthorchiidae* parasites. Although molecular methods such as PCR targeting the *ITS2* and *COX1* gene regions have been applied in Russia and Europe, published molecular data on *Opisthorchiidae* infections in Kazakhstan remain scarce. Importantly, there is limited integration of field-based epidemiological surveys with molecular differentiation of *O. felineus* and *M. bilis*, creating a critical gap in understanding their distribution, genetic variability, and role in zoonotic transmission under the “One Health” framework.

This study aimed to address the diagnostic and epidemiological challenges associated with opisthorchiasis and metorchiasis in Kazakhstan by combining parasitological and molecular approaches. Specifically, the objectives were to (i) conduct monitoring surveys of cyprinid fish from three lakes in the Akmola region to determine the prevalence and intensity of infection with *Opisthorchiidae* metacercariae, (ii) perform morphological identification of *O. felineus* and *M. bilis* larvae, (iii) develop and apply a multiplex PCR assay targeting the mitochondrial cytochrome c oxidase subunit 1 (*COX1*) gene to achieve species-level differentiation, and (iv) validate molecular identification through sequencing and phylogenetic analysis. By addressing the present lack of reliable molecular epidemiological data on *Opisthorchiidae* infections in Kazakhstan, this study seeks to provide robust tools for the differential diagnosis of *O. felineus* and *M. bilis*. Ultimately, the findings are expected to improve surveillance capacity, guide food safety measures, and contribute to national and regional control strategies for fish-borne trematodiases.

## MATERIALS AND METHODS

### Ethical approval

All stages of the research were conducted at the joint-stock company “S” scientific and technical facilities of Seifullin Kazakh Agrotechnical Research University: At the Scientific and Production Platform for Agricultural Biotechnology and the Joint Kazakh-Chinese Laboratory for Biological Safety, S. Seifullin Kazakh Agrotechnical Research University. Laboratory animals were not used in this study. All procedures involving fish were carried out in compliance with high biosafety standards and approved by the Animal Ethics Committee of the Faculty of Veterinary Medicine and Animal Husbandry Technology, S. Seifullin Kazakh Agrotechnical Research University, Astana, Kazakhstan (protocol No. 1, February 23, 2023). All protocols adhered to the International Guiding Principles for Biomedical Research [23].

### Study period and location

The research was conducted between 2021 and 2024 at the Research Platform for Agricultural Biotechnology and the accredited Joint China-Kazakhstan Laboratory of Biosafety, Faculty of Veterinary Medicine and Animal Husbandry Technology, S. Seifullin Kazakh Agrotechnical Research University, Astana, Kazakhstan.

**Figure 1:**
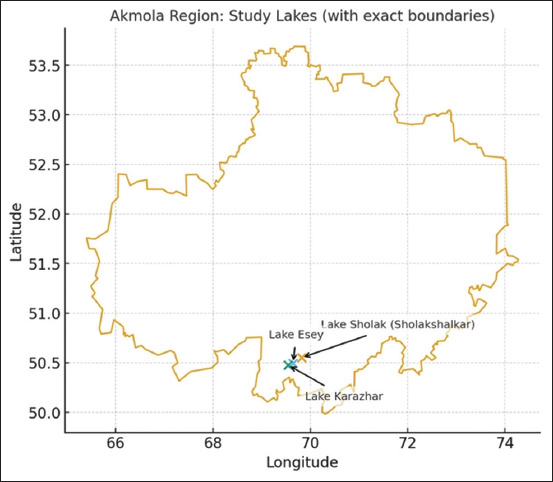
Map of the study area in Akmola region, Kazakhstan, showing the sampling sites (Lakes Sholak, Esey, and Karazhar) [Source: The map was generated by the authors using open-source data from Natural Earth and OpenStreetMap].

The study was performed in three freshwater lakes in the Akmola region of Northern Kazakhstan: Lake Sholak, Lake Esey, and Lake Karazhar (Figure 1). These lakes, part of the Nura River basin and connected to the Korgalzhyn lake system, were chosen because fish from these waters are regularly supplied to Astana’s markets. Their hydrological regime is defined by seasonal flood and groundwater replenishment, followed by partial summer isolation.

According to regional monitoring, water quality was classified as moderately polluted (saprolite index 1.8–1.85; class III). Phytoplankton biomass was ~0.018 mg/dm^3^, with ~0.10 thousand cells/cm^3^, based on 99 samples from 28 sites. Such conditions favor the development of mollusks and cyprinid fish as intermediate hosts, maintaining the circulation of *Opisthorchiidae* parasites.

### Fish collection and sampling strategies

A total of 818 freshwater fish were collected from the three lakes during summer (May–July) between 2021 and 2023. Cyprinid fish (family *Cyprinidae*) were specifically targeted during summer, when *Opisthorchis* infection peaks, to maximize metacercariae detection. Fish were captured with gill and cast nets in collaboration with local fishermen.

The sampling strategy prioritized the most commonly consumed fish species, with sample sizes stratified by species and lake. Selection was semi-randomized to ensure representation of multiple fish families. All fish were transported on ice and examined in the laboratory within 24 h.

### Parasitological study

Fish muscles were examined using the compression method. For each fish, three dorsal and two abdominal samples were taken from both sides. Muscle samples (0.2–0.5 cm thick) were placed in a MIS-7M (Laborkomplekt, Russian Federation) compressor glass and examined under a low-magnification microscope. At least 10 randomly selected fields per fish were evaluated.

Metacercariae were identified using morphological keys. Infection prevalence was calculated as the proportion of infected fish, while infection intensity was expressed as the mean number of metacercariae per infected fish ± standard error (SE).

### DNA extraction and PCR assay

For DNA extraction, a single metacercaria of each parasite type was homogenized in an Eppendorf tube (Eppendorf SE, Germany) using the standard phenol-chloroform method with proteinase K digestion and ethanol precipitation. DNA purity and concentration were measured with a NanoDrop 2000 spectrophotometer (Thermo Scientific, USA), with A260/A280 ratios of 1.8–2.0 considered acceptable. DNA was dissolved in double-distilled water (Millipore, Germany) and stored at −70°C.

PCR was performed in 25 μL reactions containing: 10× Taq buffer (Thermo Scientific) with (NH4)2SO4, 2.5 mM MgCl2, 1 U Taq DNA polymerase, 200 μM deoxynucleotide triphosphate (Thermo Scientific), 10 pmol of each primer, and 20 ng of trematode DNA template. Thermal cycling included 35 cycles of denaturation (95°C, 30 s), annealing (59°C, 40 s), and extension (72°C, 50 s).

Species-specific primers targeting the mitochondrial *COX1* gene were designed *de novo* based on GenBank references. PCR reactions were performed in triplicate for quality assurance. Positive controls (DNA of confirmed *O. felineus* and *M. bilis*) and negative controls (no-template) were included. Products were separated by electrophoresis on a 1.5% agarose gel (Lonza, Switzerland) with ethidium bromide in 1× Tris-acetate-EDTA buffer (Thermo Scientific).

A multiplex PCR assay was used to differentiate *Opisthorchis* and *Metorchis* using primers CO1nOf-F/CO1nOf-R and CO1nMb-F/CO1nMb-R.

### Sequencing and phylogenetic analysis

Amplicons were sequenced using the Sanger method and BigDye Terminator Sequencing Kit (Thermo Scientific). The same primers used for PCR were applied. Sequencing was conducted on an Applied Biosystems 3130XL genetic analyzer (Applied Biosystems, USA). Chromatograms were analyzed with Sequencing Analysis 5.2, Patch 2 (Applied Biosystems).

Two representative sequences were submitted to GenBank (accession numbers: PQ669120 and PQ669125). Sequences were compared with GenBank entries using the basic local alignment search tool, confirming >99% similarity. Phylogenetic analysis was performed in MEGA 11 software (Pennsylvania State University, USA). Evolutionary history was inferred using the maximum composite likelihood method, with distances expressed as base substitutions per site. The Closest Neighbor Interchange algorithm was used for tree searching, and Neighbor-Joining was applied to construct initial trees. Phylogenetic trees were generated with 1000 bootstrap replicates using the Tamura–Nei substitution model.

### Quality control and replicates

All PCR analyses were performed in duplicate or triplicate to ensure reproducibility. Infection intensity and prevalence were calculated using standard methodology [24]. Strict laboratory protocols were followed to prevent contamination, with separate areas designated for DNA extraction, PCR setup, and post-PCR analysis.

### Statistical analysis

Prevalence was calculated as the number of infected fish divided by the total number examined per species and lake. Infection intensity was expressed as mean ± SE. Comparisons of prevalence between species and lakes were performed using Chi-square tests, with p ≤ 0.05 considered statistically significant. Prevalence values were reported with 95% confidence intervals. Statistical analyses were performed using GraphPad Prism 3.0 (GraphPad Software, Inc., San Diego, USA).

## RESULTS

### Detection of *Opisthorchiidae* metacercariae

**Figure 2:**
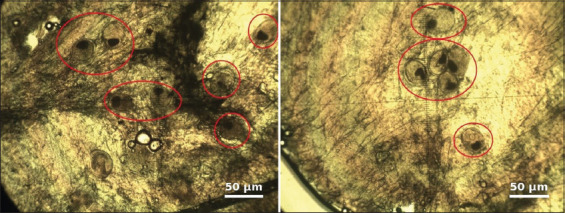
Microscopy of a compression preparation from the muscle tissue of an infected fish (ide, Lake Sholak), 10× magnification. Metacercariae indicated by red circles.

During the parasitological examination of the musculature of 818 cyprinid fish specimens, metacercariae belonging to the family *Opisthorchiidae* were detected. *Opisthorchis* metacercariae were characterized by an oval-shaped cyst containing a folded helminth larva (Figure 2). At low infection intensities, most larvae were localized in the dorsal musculature; however, at high infection intensities, as observed in ide, metacercariae were distributed throughout all muscle tissues.

**Figure 3:**
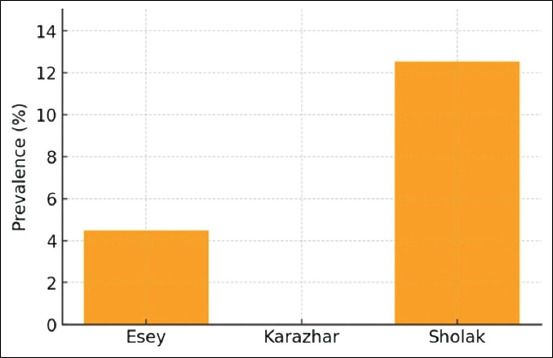
Prevalence of *Opisthorchiidae* metacercariae in fish from three lakes (Sholak, Esey, and Karazhar) in the Akmola region, Kazakhstan.

The prevalence of *Opisthorchiidae* metacercariae varied across lakes (Figure 3). Notably, all cyprinid fish examined from Lake Sholak were infected, whereas no *Opisthorchis* or *Metorchis* pathogens were detected among cyprinid species in Lake Karazhar. The prevalence and intensity of infection are presented in Table 1.

**Table 1:** Species composition of examined cyprinid fish samples from Lakes Sholak, Esey, and Karazhar.

No.	Sampling location	Fish species	Total examined samples (n)	Number of infected samples (n)	Prevalence (95% CI) (%)	Main intensity ± SE
1	Sholak	Ide	160	53	26.3–40.7	33.1 ± 3.7
		Bream	107	4	1.5–9.3	3.7 ± 0.018
		Roach	363	22	4.0–9.0	6.1 ± 0.013
2	Esey	Ide	41	3	2.5–24.2	7.3 ± 0.041
		Chebak	12	-	-	-
		Crucian carp	13	-	-	-
		Bream	1	-	-	-
3	Karazhar	Ide	96	-	-	-
		Crucian carp	15	-	-	-
		Bream	10	-	-	-

CI = Confidence interval, SE = Standard error.

### Prevalence and intensity across fish species

According to the parasitological examination, ide exhibited the highest prevalence of infection (40.4%), likely due to its natural susceptibility to *Opisthorchiidae* [24]. Bream and roach demonstrated nearly identical infection intensities in Lake Sholak; however, prevalence was 2.4% higher in roach. The overall prevalence of infection in Lake Sholak reached 42.9%, which is significant considering that only cyprinid species were examined.

In Lake Esey, only ide was infected, showing a prevalence of 7.3% and an infection intensity of 3.3%. No larvae were detected in the other examined species. In Lake Karazhar, none of the 121 examined fish specimens carried parasites.

### Morphological identification

**Figure 4:**
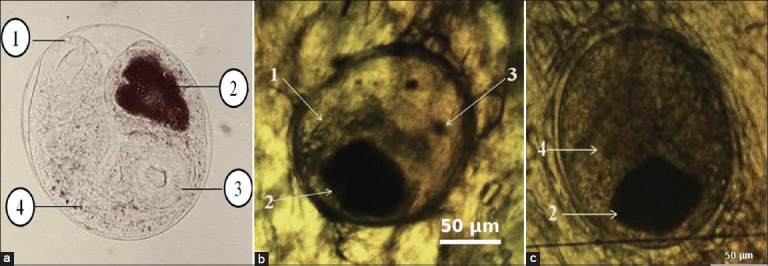
Morphological analysis of *Opisthorchiidae* larvae isolated from fish muscle tissue: 1 – intestine; 2 – excretory bladder; 3 – oral sucker; and 4 – ventral sucker. (a) According to the identification key by Bykhovskaya-Pavlovskaya *et al*. [25], (b and c) *Opisthorchiidae* from cyprinid fish.

Morphological identification was performed by microscopic examination of muscle sections using the identification key of Bykhovskaya-Pavlovskaya *et al*. [25]. All examined muscle tissues contained the characteristic round capsules (Figure 4) [25].

The metacercariae of *O. felineus* measured 160–190 μm in diameter, whereas those of *M. bilis* were slightly smaller (140–180 μm). Both species exhibited a round or slightly oval shape with a double-layered cyst wall. A distinct difference was noted in the prominence of the oral sucker (3) and the intestine (1) in *M. bilis*. No other consistent morphological parameters distinguished the two species. Compared with the identification key, both parasites showed similar morphological features. Due to these subtle differences, a reliable distinction between *O. felineus* and *M. bilis* is challenging and requires highly skilled personnel [26, 27]. The isolated metacercariae were identified as *O. felineus* and *M. bilis* larvae.

### Molecular differentiation by PCR

PCR amplification using the newly designed COX1 primers yielded species-specific bands for *O. felineus* and *M. bilis*. The primer sequences were CO1nOf-F (TTGGA-ATGAT-TAGTC-ATGTT-TGTAC-G) and CO1nOf-R (CCCCA-CCTAT-AGTAA-AAAGC-ACTAT). The annealing temperature was 54°C. The expected product size was 307 bp for *O. felineus* and 252 bp for *M. bilis*. These differences in molecular weight allowed reliable species differentiation. Positive and negative controls confirmed assay specificity and ruled out contamination.

**Figure 5:**
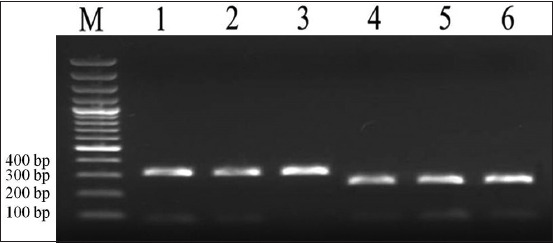
Result of multiplex polymerase chain reaction for *Opisthorchis* felineus and Metorchis bilis: M, marker; lanes 1–3, *O. felineus* (1, ide, Lake Sholak; 2, bream, Lake Sholak; 3, roach, Lake Sholak); lanes 4–6, *M. bilis* (ide, Lake Sholak).

Species identification was further verified using multiplex PCR with primers targeting the mitochondrial cluster. Amplification with CO1nOf-F/R and CO1nMb-F/R produced species-specific bands of 307 bp and 252 bp, respectively (Figure 5).

### Sequencing and phylogenetic confirmation

The PCR products were sequenced, and species identity was confirmed with >99% similarity to GenBank reference sequences. Two representative sequences were deposited in GenBank: PQ669120 (*O. felineus*) and PQ669125 (*M. bilis*). These primers thus provided precise identification of the causative agents of opisthorchiasis and metorchiasis.

**Figure 6:**
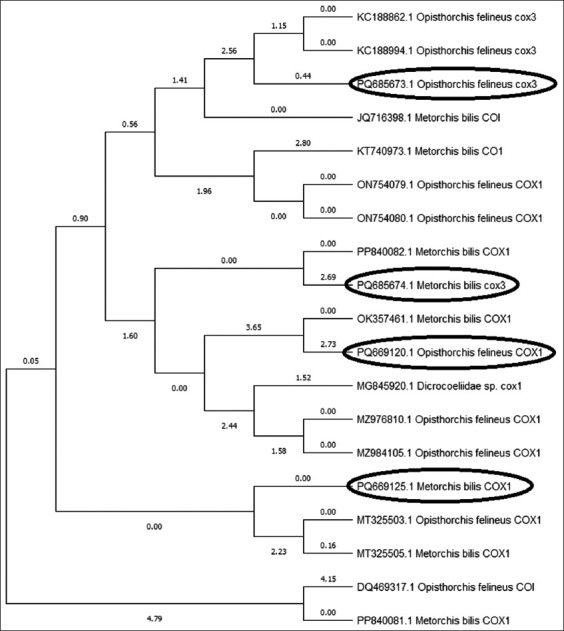
Phylogenetic tree constructed for *Opisthorchis felineus* and *Metorchis bilis* based on mitochondrial *COX1* and *COX3* genes. Sequenced samples are highlighted in black.

Phylogenetic analysis of reference sequences demonstrated strong bootstrap support (>95%), confirming the molecular differentiation between the two species (Figure 6).

### Phylogenetic relationships of isolates

The results showed that sample PQ685673.1 (*COX3* gene) clustered with other *O. felineus* sequences (KC188862.1 and KC188994.1), demonstrating a high degree of relatedness and confirming its taxonomic assignment, with node support values ranging from 1.15 to 2.56. Sample PQ685674.1 (*COX3* gene) clustered with *M. bilis* sequences, including OK357461.1, with a node support value of 2.69.

Sample PQ669120.1 (*COX1* gene) grouped within the *O. felineus* cluster alongside isolates ON754079.1 and ON754080.1, with a node support value of 2.73. Sample PQ669125.1 (*COX1* gene) clustered with *M. bilis* sequences, such as MT325505.1, although phylogenetic diversity suggested possible intraspecific divergence or geographic variability within *M. bilis* populations.

### Summary of findings

All specimens obtained in the present study were reliably identified, confirming the taxonomic validity of using mitochondrial *COX1* and *COX3* genes for differentiating trematodes of the genera *Opisthorchis* and *Metorchis*. The generated molecular data provide a foundation for future genetic and epidemiological studies of these parasites in Kazakhstan.

## DISCUSSION

### Epidemiological insights

Our study provides new epidemiological data on *Opisthorchiidae* infections in freshwater fish from Akmola, Kazakhstan. We confirmed the presence of *O. felineus* and *M. bilis* in three lakes using a combination of field sampling and molecular analyses, with variable prevalence across sites. The observed prevalence rates (19.7%–35.2%) are consistent with reports from neighboring countries, such as Kyrgyzstan (20%–30%) [28] and Russia (>40% in cyprinid fish) [29], while some regions of China report lower levels (<15%) [30]. These findings emphasize the need for coordinated regional monitoring strategies across Eurasia.

### Public health significance and risk factors

*Opisthorchiidae* infections remain of major public health importance because of their zoonotic potential. In Kazakhstan, opisthorchiasis continues to pose a serious problem, with human prevalence rates in hyperendemic regions ranging between 5% and 30% [31]. The integration of veterinary, medical, and ecological surveillance within the One Health framework is crucial for enhancing fish inspection systems, increasing consumer awareness, and reducing zoonotic transmission risks.

Ecological factors, including hydrology, lake depth, and connectivity with rivers, appear to influence the variation in the prevalence of infection among the lakes under study. In fish belonging to the *Cyprinidae* family, *Opisthorchis* infestations peak in the summer due to increased cercariae shedding by *Bithynia* snails. This seasonal trend results in a higher accumulation of metacercariae in fish tissues, thereby increasing the risk of human infection. The role of snail vectors (*Bithynia* spp.) in sustaining transmission in Kazakhstan remains poorly characterized and warrants further study [32]. In addition, cultural practices, particularly the consumption of raw or undercooked fish, continue to represent a major risk factor for human infection.

### Molecular and genomic contributions

Our findings contribute to the growing body of genomic knowledge on *O. felineus* at the molecular level. The NCBI database currently contains 286 protein and 390 nucleotide sequences of this parasite, and its nuclear genome (684 Mbp) encodes 11,455 proteins [33]. Its mitochondrial genome is the shortest among trematodes (14,277 bp), comprising 35 genes (2 ribosomal ribonucleic acids, 22 transfer ribonucleic acids, and 12 proteins) [27]. Polymorphisms within the mitochondrial genome, along with conserved microRNA markers (miR-281 and miR-7), represent promising molecular tools for parasite biology, diagnostics, and molecular epidemiology [34].

Our phylogenetic analysis, based on the *COX1* gene, confirmed a close evolutionary relationship between *O. felineus* and *O. lobatus*, while clearly separating them from *O. viverrini* and showing a distinct clustering of *Metorchis* spp. These results highlight the taxonomic validity of separating the *Opisthorchis* and *Metorchis* genera and underscore the utility of mitochondrial markers for species identification.

### Diagnostic advances

The multiplex PCR primers developed in this study were efficient for differentiating *O. felineus* from *M. bilis*, providing a valuable diagnostic tool for epidemiological surveys. However, only two sequences were deposited due to resource limitations, which restricted the breadth of phylogenetic insights. Expanding molecular characterization to a larger number of isolates from diverse regions will improve the phylogeographic resolution of *Opisthorchiidae* parasites in Kazakhstan.

## CONCLUSION

This study provides the first molecular confirmation of *O. felineus* and *M. bilis* in freshwater fish from the Akmola region of Kazakhstan. Across parasitological examination of 818 cyprinid fish, we detected a variable prevalence of *Opisthorchiidae* metacercariae, with ide exhibiting the highest infection rate (40.4%) and Lake Sholak showing the greatest overall prevalence (42.9%). In contrast, no *Opisthorchiidae* larvae were identified in fish from Lake Karazhar. Morphological analyses confirmed the presence of *Opisthorchiidae* cysts but revealed overlapping characteristics between species, making microscopic differentiation unreliable. Molecular approaches, particularly the newly developed multiplex PCR assay targeting the *COX1* gene, successfully distinguished *O. felineus* (307 bp) from *M. bilis* (252 bp), with sequencing confirming >99% identity to GenBank references. Two representative sequences were deposited in GenBank, and phylogenetic analysis validated the taxonomic separation of the two species.

The findings highlight the zoonotic risk associated with consuming raw or undercooked fish from local water bodies and emphasize the need for stronger food safety measures. The multiplex PCR assay developed in this study provides a rapid, reliable, and cost-effective diagnostic tool for differential identification of *Opisthorchiidae* species, with direct applications in epidemiological surveys, veterinary inspection, and public health monitoring. Integrating such molecular tools into surveillance systems will improve fish safety inspections, enhance early detection of infections, and support prevention strategies for fish-borne trematodiases.

This work is strengthened by its integration of field-based parasitological surveys with molecular diagnostics, providing robust evidence of the circulation of *O. felineus* and *M. bilis* in Kazakhstan. It also contributes new genetic data to international databases, enhancing resources for future *Opisthorchiidae* research. However, the study was limited to three lakes and summer sampling, resulting in only a small number of isolates being sequenced. Additionally, the role of snail intermediate hosts was not investigated, which may limit the broader epidemiological conclusions.

Future research should expand sampling to additional water bodies and include multiple seasons to capture temporal variation in prevalence. Investigations into snail intermediate hosts are necessary to gain a better understanding of transmission dynamics. Longitudinal molecular surveys, coupled with the development of field-applicable diagnostic tools and expanded sequencing of isolates from different regions, will refine phylogeographic and evolutionary insights into *Opisthorchiidae* parasites.

In conclusion, this study demonstrates the importance of combining parasitological and molecular methods for the accurate identification of *O. felineus* and *M. bilis*. The results provide valuable epidemiological insights and practical diagnostic tools for Kazakhstan, with wider implications for Eurasian surveillance of fish-borne trematodiases. Strengthening regional monitoring under the One Health framework is essential to mitigate zoonotic risks, safeguard public health, and contribute to global efforts against neglected foodborne parasitic infections.

## AUTHORS’ CONTRIBUTIONS

ABB: Collected fish samples, performed laboratory analysis, and drafted the manuscript. AVK: Interpreted the results, performed statistical analysis, and reviewed and edited the manuscript. KBM: Performed laboratory analysis and data curation. All authors have read and approved the final manuscript.

## References

[1] Zaparina O.G, Kapushchak Y.K, Lishai E.A, Hong S.J, Sripa B, Pakharukova M.Y (2024). Species-specific renal and liver responses during infection with food-borne trematodes *Opisthorchis felineus*, *Opisthorchis viverrini*, or *Clonorchis sinensis*. PLoS One.

[2] Petney T.N, Andrews R.H, Saijuntha W, Wenz-Mücke A, Sithithaworn P (2013). The zoonotic, fish-borne liver flukes *Clonorchis sinensis*, *Opisthorchis felineus* and *Opisthorchis viverrini*. Int. J. Parasitol.

[3] Fedorova O.S, Fedorova M.M, Sokolova T.S, Golovach E.A, Kovshirina Y.V, Ageeva T.S, Kovshirina A.E, Kobyakova O.S, Ogorodova L.M, Odermatt P (2019). The role of *Bithynia* snails in the transmission of *Opisthorchis felineus* in Western Siberia. Parasitol. Int.

[4] Kovner A, Kapushchak Y, Hadieva E, Persidskij M, Pakharukova M (2025). IgA nephropathy is associated with *Opisthorchis felineus* liver fluke infection:Retrospective 5-year analysis of human kidney samples. Trop. Med. Int. Health.

[5] Jaume-Ramis S, Martínez-Ortí A (2021). Iberian distribution of the freshwater snail genus Bithynia Leach, 1818 (*Mollusca*:*Truncatelloidea*), vector of opisthorchiasis and metorchiasis. Acta Parasitol.

[6] Tsukanov V.V, Vasyutin A.V, Tonkikh J.L (2024). Parasites of the liver:A global problem. World J. Gastroenterol.

[7] Ponomarev D, Lvova M, Mordvinov V, Chidunchi I, Dushkin A, Avgustinovich D (2025). Anti-*Opisthorchis felineus* effects of artemisinin derivatives:An *in vitro* study. Acta Trop.

[8] Akilov R.T (2021). Epidemiology of opisthorchiasis in Eastern Kazakhstan. Trop. Med. Infect. Dis.

[9] Sripa B, Yurlova N, Suwannatrai A, Serbina E, Tangkawattana S, Sayasone S, Varnakovida P (2025). Potential impact of climate change on *Opisthorchis viverrini* and *Opisthorchis felineus* transmission in Eurasia. Acta Trop.

[10] Chai J.Y, Murrell K.D, Lymbery A.J (2005). Foodborne intestinal flukes in Southeast Asia. Korean J. Parasitol.

[11] Xiang L, Jian D, Xiaoli Z, Xueli Z, Xu J, Rui C, Yang C, Yifan S, Jie W, Yu Z, Jianping C, Su H (2024). MicroRNAs in opisthorchiids and their definitive hosts:Current status and perspectives. Mol. Biochem. Parasitol.

[12] Pozio E (2008). Epidemiology and control of foodborne parasitic zoonoses in the European Union. Parasitol. Res.

[13] Avgustinovich D.F, Chadaeva I.V, Kizimenko A.V, Kovner A.V, Bazovkina D.V, Ponomarev D.V, Evseenko V.I, Naprimerov V.A, Lvova M.N (2025). The liver-brain axis under the influence of chronic *Opisthorchis felineus* infection combined with prolonged alcoholization in mice. Vavilovskii Zhurnal Genet. Breed.

[14] Keiser J, Utzinger J (2009). Food-borne trematodiases. Clin. Microbiol. Rev.

[15] Müller B, Schmidt J, Mehlhorn H (2007). PCR diagnosis of infections with different species of *Opisthorchiidae* using a rapid clean-up procedure for stool samples and specific primers. Parasitol. Res.

[16] Bryusentsov I.I, Katokhin A.V, Sakharovskaya Z.V, Sazonov A.E, Ogorodova L.M, Fedorova O.S, Kolchanov N.A, Mordvinov V.A (2010). DNA diagnosis of mixed invasions of *Opisthorchis felineus* and *Metorchis bilis* by polymerase chain reaction. Med. Parazitol.

[17] Pauly A, Schuster R, Steuber S (2003). Molecular characterization and differentiation of opisthorchiid trematodes of the species *Opisthorchis felineus* (Rivolta, 1884) and *Metorchis bilis* (Braun, 1790) using polymerase chain reaction. Parasitol. Res.

[18] Pakharukova M.Y, Savina E, Ponomarev D.V, Gubanova N.V, Zaparina O, Zakirova E.G, Cheng G, Tikhonova O.V, Mordvinov V.A (2023). Proteomic analysis of *Opisthorchis felineus* excretory-secretory products reveals potential diagnostic biomarkers and therapeutic targets. J. Proteomics.

[19] Pakharukova M.Y, Mordvinov V.A, Yurlova N.I, Katokhin A.V (2012). Sequence variability in the mitochondrial cytochrome c oxidase I gene in *Opisthorchis felineus* from Western Siberia. Parasitol. Int.

[20] Bekenova A.B, Smagulova A, Katokhin A, Borovikov S, Kiyan V (2020). Molecular differential diagnosis between *Opisthorchis felineus* and *Metorchis bilis*. Adv. Anim. Vet. Sci.

[21] Kiyan V.S, Bulashev A.K, Katokhin A.V (2018). *Opisthorchis felineus* and *Metorchis bilis* metacercariae in cyprinid fish *Leuciscus idus* in Nura-Sarysu River, Kazakhstan. Korean J. Parasitol.

[22] Council for International Organizations of Medical Sciences (CIOMS). International Council for Laboratory Animal Science (ICLAS) (2012). International Guiding Principles for Biomedical Research Involving Animals. CIOMS/ICLAS.

[23] Bobrek K, Gaweł A, Piasecki T, Bobusia K, Mazurkiewicz M (2012). Extensiveness and intensity of invasion of intestinal parasites in flocks of racing pigeons in the south of Poland. Acta Sci. Pol. Med. Vet.

[24] Beer S.A (2005). Biology of the Causative Agent of Opisthorchiasis.

[25] Bykhovskaya-Pavlovskaya I.E, Gusev A.V, Lubinina M.N, Izyumova N.A, Smirnova T.S, Sokolovskaya I.L, Shtein G.A, Shulman S.S, Epstein V.M (1962). Key to the parasitic helminths of freshwater fish of the USSR.

[26] Tatonova Y.V, Besprozvannykh V.V (2019). Molecular identification of trematodes of the genus Metorchis in Russia. Parasites Vectors.

[27] Mordvinov V.A, Ershov N.I, Zaparina O.G, Pakharukova M.Y (2020). Genomics and proteomics of the liver fluke *Opisthorchis felineus. Vavilovskii Zhurnal Genet*. Breed.

[28] Tidman R, Kanankege K.S.T, Bangert M, Abela-Ridder B (2023). Global prevalence of four neglected foodborne trematodes targeted for control by WHO:A scoping review to highlight the gaps. PLoS Negl. Trop. Dis.

[29] Simakova A.V, Chitnis N, Babkina I.B, Fedorova O.S, Fedotova M.M, Babkin A.M, Khodkevich N.E (2021). Abundance of *Opisthorchis felineus* metacercariae in cyprinid fish in the middle Ob River basin (Tomsk region, Russia). Food Waterborne Parasitol.

[30] Qian M.B, Utzinger J, Keiser J, Zhou X.N (2016). Clonorchiasis. Lancet.

[31] Aubakirov M.Z, Abdybekova A.M, Khassanova M.A, Issabayev A.Z, Kaumenov N.S, Tegza A.A, Sapa V.A, Domatsky V.N, Erenko E.N, Namazbai K.N (2022). Epizootology and epidemiology of opisthorchiasis in northern Kazakhstan. Open J. Biol. Sci.

[32] Fedorova O.S, Fedorova M.M, Zvonareva O.I, Mazeina S.V, Kovshirina Y.V, Sokolova T.S, Golovach E.A, Kovshirina A.E, Konovalova U.V, Kolomeets I.L, Gutor S.S, Petrov V.A, Hattendorf J, Ogorodova L.M, Odermatt P (2020). Opisthorchis felineus infection, risk, and morbidity in rural Western Siberia, Russian Federation. PLoS Neglected Tropical Diseases.

[33] Ershov N.I, Mordvinov V.A, Prokhortchouk E.B, Pakharukova M.Y, Gunbin K.V, Ustyantsev K, Genaev M.A, Blinov A.G, Mazur A, Boulygina E, Tsygankova S, Khrameeva E, Chekanov N, Fan G, Xiao A, Zhang H, Xu X, Yang H, Solovyev V, Lee S.M.Y, Liu X, Afonnikov D.A, Skryabin K.G (2019). New insights from *Opisthorchis felineus* genome:Update on genomics of the epidemiologically important liver flukes. BMC Genomics.

[34] Li X, Ding J, Zhang X, Zhang X, Jiang X, Chen R, Cheng Y, Sun Y, Wan J, Zhang Y, Cao J, Han S (2024). MicroRNAs in opisthorchiids and their definitive hosts:Current status and perspectives. Mol. Biochem. Parasitol.

